# Does early life exposure to exogenous sources of reactive oxygen species (ROS) increase the risk of respiratory and allergic diseases in children? A longitudinal cohort study

**DOI:** 10.1186/s12940-022-00902-7

**Published:** 2022-10-03

**Authors:** Teresa To, Emilie Terebessy, Jingqin Zhu, Kimball Zhang, Pascale SJ Lakey, Manabu Shiraiwa, Marianne Hatzopoulou, Laura Minet, Scott Weichenthal, Sharon Dell, Dave Stieb

**Affiliations:** 1 Dalla Lana School of Public Health, University of Toronto, Toronto, Canada; 2 Child Health Evaluative Sciences, Research Institute, The Hospital for Sick Children, Toronto, Canada; 3 ICES, Ontario, Canada; 4 Department of Chemistry, University of California Irvine, Irvine, USA; 5 Department of Civil and Mineral Engineering, University of Toronto, Toronto, Canada; 6 Department of Civil Engineering, University of Victoria, Victoria, Canada; 7 Department of Epidemiology, Biostatistics and Occupational Health, McGill University, Montreal, Canada; 8 Water and Air Quality Bureau, Health Canada, Ottawa, Canada; 9 Department of Pediatrics, Faculty of Medicine, University of British Columbia, Vancouver, Canada; 10 Pediatric Respiratory Medicine, Provincial Health Services Authority, BC Children’s Hospital, Vancouver, Canada; 11 Environmental Health Science and Research Bureau, Health Canada, Ottawa, Canada

**Keywords:** Reactive oxygen species, Asthma, Allergic rhinitis, Eczema, Air pollution, Early life exposures

## Abstract

**Background:**

Excess reactive oxygen species (ROS) can cause oxidative stress damaging cells and tissues, leading to adverse health effects in the respiratory tract. Yet, few human epidemiological studies have quantified the adverse effect of early life exposure to ROS on child health. Thus, this study aimed to examine the association of levels of ROS exposure at birth and the subsequent risk of developing common respiratory and allergic diseases in children.

**Methods:**

1,284 Toronto Child Health Evaluation Questionnaire (T-CHEQ) participants were followed from birth (born between 1996 and 2000) until outcome, March 31, 2016 or loss-to-follow-up. Using ROS data from air monitoring campaigns and land use data in Toronto, ROS concentrations generated in the human respiratory tract in response to inhaled pollutants were estimated using a kinetic multi-layer model. These ROS values were assigned to participants’ postal codes at birth. Cox proportional hazards regression models, adjusted for confounders, were then used to estimate hazard ratios (HR) with 95% confidence intervals (CI) per unit increase in interquartile range (IQR).

**Results:**

After adjusting for confounders, iron (Fe) and copper (Cu) were not significantly associated with the risk of asthma, allergic rhinitis, nor eczema. However, ROS, a measure of the combined impacts of Fe and Cu in PM_2.5_, was associated with an increased risk of asthma (HR = 1.11, 95% CI: 1.02–1.21, p < 0.02) per IQR. There were no statistically significant associations of ROS with allergic rhinitis (HR = 0.96, 95% CI: 0.88–1.04, p = 0.35) and eczema (HR = 1.03, 95% CI: 0.98–1.09, p = 0.24).

**Conclusion:**

These findings showed that ROS exposure in early life significantly increased the childhood risk of asthma, but not allergic rhinitis and eczema.

## Background

Studies have shown that excess reactive oxygen species (ROS) can cause oxidative stress injuring cells and tissues leading to adverse health effects in the respiratory tract.[1–3] Both endogenous and exogenous sources of ROS (e.g., cigarette smoke, air pollution) can play an important role in the pathogenesis and worsening of various inflammatory conditions such as asthma.[4–8] Lakey et al. found that in highly polluted environments, fine particulate matter (PM_2.5_) containing redox-active transition metals, quinones, and secondary organic aerosols can increase ROS concentrations in the epithelial lining fluid (ELF) to levels characteristic of respiratory diseases.[1] Children are more engaged in physical activities, spend more time outdoors, breathe more rapidly than adults (therefore absorb more pollutants), and thus are more vulnerable to the effects of ROS. However, to date, few human epidemiological studies have quantified the adverse effect of early life exposure to ROS on child health. To fill the knowledge gap, this current study examines the association of levels of ROS exposure at birth and the subsequent risk of developing common childhood respiratory and allergic diseases, namely allergic rhinitis and eczema, in children.

## Methods

### Study population and data linkage

The Toronto Child Health Evaluation Questionnaire (T-CHEQ) study established a population-based cohort of 5,619 grades one and two (aged 5 to 9 years) Toronto school children in 2006 using modified International Study of Asthma and Allergies in Childhood (ISAAC) methodology.[9] Parent-reported lifetime asthma prevalence in this population of Toronto children was high (16.1%).[9] In the 2006 T-CHEQ survey, participants were asked to provide permission/consent to use their unique Health Card Number (HCN) for data linkage in future research. The 1,284 consented T-CHEQ participants who were born in Ontario were linked to the Ontario population-based health administrative databases to ascertain both their home address histories in Ontario (to assign exposures) and their health services claims (to measure outcomes) from birth to outcome, end of follow-up (March 31, 2016), or loss-to-follow-up. This linked cohort formed the T-CHEQ Birth Cohort for this study. With this linked cohort, we aimed to measure the association of common childhood respiratory (asthma) and allergic diseases (allergic rhinitis and eczema) with early life exposures to ROS.

### Measures

#### Outcome measures

Children and youths were classified as having incident asthma by International Classification of Disease codes (ICD-9: 493 and ICD-10: J45, J46) if they had: ≥ 1 asthma hospital admission or ≥ 2 asthma Ontario Health Insurance Plan claims for physician services in two consecutive years (where the first claim is considered the diagnosis date). This case definition has been validated with 84% sensitivity and 76% specificity.[10, 11] Allergic rhinitis (ICD-9: 477 and ICD-10: J301-J304) and eczema (ICD-9: 691.8 and ICD-10: L20) were identified by physicians with any health services use for these conditions by their respective ICD codes. The T-CHEQ Birth Cohort was linked to Ontario health administrative databases housed at ICES (formerly known as the Institute for Clinical Evaluative Sciences) using an encrypted unique HCN given to all Ontario residents. The health administrative databases used in this study included the National Ambulatory Care Reporting System (emergency department visits), Canadian Institute for Health Information Discharge Abstract Database (hospital admissions), Ontario Health Insurance Plan Claims Database (physician outpatient claims), to capture health services use. All children in the T-CHEQ Birth Cohort were followed prospectively from birth to their index date of outcomes of interest, death, end-of-study, or loss-to-follow-up.

#### ROS exposures

The ROS data were generated by Weichenthal et al. [12] from large-scale air monitoring campaigns during summer 2016 and winter 2017 in Toronto, Canada. Land use data around each monitoring site were used to develop predictive models for iron (Fe), copper (Cu), and their estimated combined impact on ROS generation. Weichenthal et al. used the KM-SUB-ELF (kinetic multilayer model of surface and bulk chemistry in the lung epithelial lining fluid) to estimate ROS concentrations generated in the human respiratory tract in response to inhaled pollutants including Fe and Cu in PM_2.5_. [12] The model provides a useful means of estimating ROS values and the combined health impacts of Fe and Cu in PM_2.5_. Annual ROS values were then assigned to residential postal codes of the participants’ year of birth.

#### Covariates

There were three domains of parent-reported covariates collected by the 2006 T-CHEQ survey: child, parental/demographics, and home environmental factors. Child factors included: age, sex, low birth weight (< 2500 g), prematurity (child born within three weeks of due date), breastfeeding, and enrollment in childcare. In this first domain, age was a continuous variable while the remaining variables had outcomes categorized as yes/no and male/female for sex. Parental/demographic factors included: parental asthma and atopy, parental education level, and income adequacy. In this second domain, parental asthma and atopy were variables with yes/no outcomes. Parental education levels were based on highest level of education attained and categorized as: Less than or some college or university; college diploma; university degree. Income adequacy levels were based on household income and categorized as: Lowest income adequacy; lower middle income adequacy; upper middle income adequacy. Home environmental factors included: crowding (number of people living in household) and home exposures during first year of life (use of gas to cook or heat, exposure to environmental tobacco smoke, pets, cockroaches, and mould in the home). This last domain consisted of variables with yes/no outcomes, except for crowding which was a discrete variable categorized as 4 people or ≥ 5 people in household.

### Statistical analysis

The relationship between outcomes of interest and ROS exposure was examined using the Cox proportional hazards (PH) regression model. All hazard ratios (HR) were presented with 95% confidence intervals (CI), and were calculated per ROS or pollutant interquartile range (IQR). The Cox PH regression models were repeated for three health outcomes: asthma, allergic rhinitis, and eczema. All covariates within the three domains (child, parental/demographics, and home environmental factors) were included in the multivariable Cox PH regression models to adjust for potential confounding. Plots of the Martingale-based residuals were examined to test if the proportionality assumption of the Cox PH models was met. All analyses were carried out using SAS Enterprise guide 9.4 (SAS Institute Inc., Cary, NC).

## Results

**Fig. 1:**
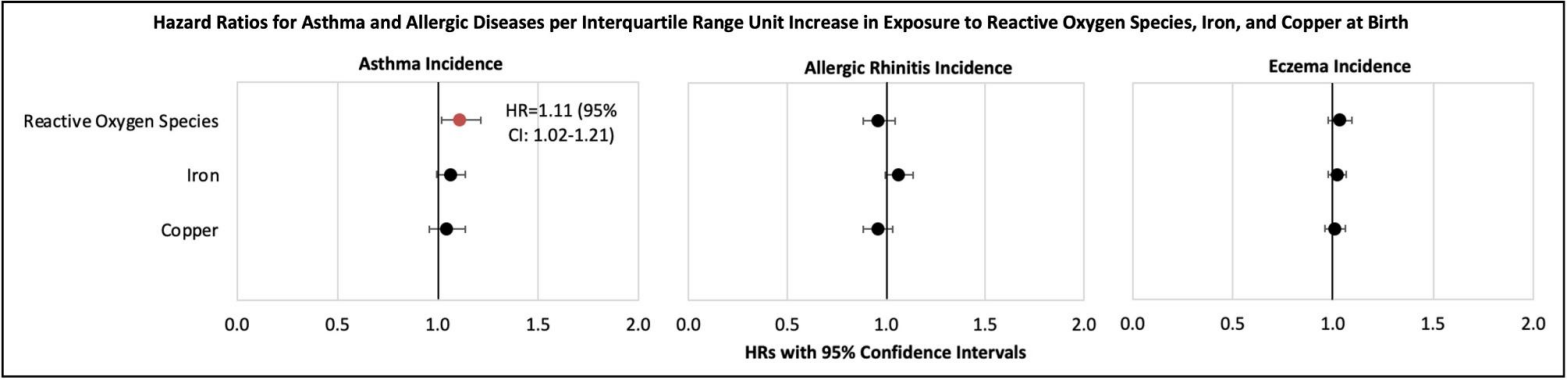
Hazard ratios for asthma, allergic rhinitis, and eczema based on multivariable Cox proportional hazard regressions* Abbreviations: HR = hazard ratio; CI = confidence interval. *Note: All models for 1,284 participants in this study were adjusted for 16 covariates: 6 child factors, 4 parental/demographic factors and 6 home environmental factors. Child factors included: age, sex, low birth weight (<2,500g), prematurity (child born within three weeks of due date), breastfeeding, and enrollment in childcare. Parental/demographic factors included: parental asthma and atopy, parental education level, and income adequacy. Home environmental factors included: crowding (number of people living in household) and home exposures during first year of life (use of gas to cook or heat, exposure to environmental tobacco smoke, pets, cockroaches, and mould in the home).

In our study population, the annual levels of ROS, Fe, and Cu were 58.4 nM (± 12.6 standard deviation [SD]), 1.45 ng/m3 (± 0.35 SD), and 0.07 ng/m3 (± 0.02 SD), respectively. Among the 1,284 study participants, 365 (28.4%) had asthma, 510 (39.7%) had allergic rhinitis, and 956 (74.5%) had eczema. The mean ages of participants were 6.7 years (± 0.65 SD). 680 (53.0%) participants were male and 604 (47.0%) were females. The mean ages of participants at outcomes were 3.33 (± 3.60 SD) for asthma, 6.31 (± 4.41 SD) for allergic rhinitis, and 2.89 (± 4.12 SD) for eczema. More male participants had asthma (227 or 62.0%), allergic rhinitis (295 or 57.7%), and eczema (520 or 54.3%) compared to female participants. Consistent with the literature, our study also found common risk factors that increased the risk of childhood asthma, allergic rhinitis, and eczema: being male, with parental history of asthma, and lower middle income adequacy (Table 1). Results (Fig. 1) from the multivariable Cox PH regressions showed that after adjusting for confounders, Fe and Cu were not significantly associated with the risk of asthma, allergic rhinitis, nor eczema. However, ROS, which measures the combined impacts of Fe and Cu in PM_2.5_, was associated with an 11% increase in the risk of asthma (HR = 1.11, 95% CI: 1.02–1.21, p < 0.02) per IQR. The associations of ROS with allergic rhinitis (HR = 0.96, 95% CI: 0.88–1.04, p = 0.35) and eczema (HR = 1.03, 95% CI: 0.98–1.09, p = 0.24) were not statistically significant.

**Table 1:** Hazard ratios for childhood diseases per interquartile range unit increase in exposures at birth*

Factors	Asthma						Allergic Rhinitis						Eczema					
	**HR**		**95% CI**				**HR**		**95% CI**				**HR**		**95% CI**			
***Exposure***																		
Reactive Oxygen Species	1.11	(	1.02	-	1.21	)	0.96	(	0.88	-	1.04	)	1.03	(	0.98	-	1.09	)
Iron	1.06	(	0.99	-	1.14	)	1.06	(	0.99	-	1.14	)	1.02	(	0.98	-	1.06	)
Copper	1.04	(	0.96	-	1.14	)	0.95	(	0.88	-	1.03	)	1.01	(	0.96	-	1.06	)
***Child factors***																		
*Age group*																		
7 years	0.95	(	0.76	-	1.18	)	1.03	(	0.86	-	1.24	)	1.00	(	0.88	-	1.15	)
8–9 years	1.11	(	0.78	-	1.57	)	0.82	(	0.59	-	1.15	)	1.15	(	0.91	-	1.44	)
Male sex	1.62	(	1.31	-	2.00	)	1.28	(	1.07	-	1.53	)	1.15	(	1.01	-	1.31	)
Low birthweight (< 2500 g)	1.30	(	0.82	-	2.04	)	0.93	(	0.60	-	1.43	)	1.07	(	0.79	-	1.45	)
Breastfeeding	0.64	(	0.45	-	0.89	)	0.81	(	0.60	-	1.10	)	1.00	(	0.79	-	1.27	)
Enrollment in childcare	1.04	(	0.82	-	1.32	)	0.91	(	0.75	-	1.11	)	1.14	(	0.98	-	1.32	)
Child born within three weeks of due date	1.27	(	0.82	-	1.94	)	0.93	(	0.62	-	1.40	)	0.94	(	0.71	-	1.24	)
***Parental/demographic factors***																		
Parental history of asthma	1.37	(	1.05	-	1.80	)	1.29	(	1.02	-	1.63	)	0.98	(	0.82	-	1.16	)
Parental history atopy	1.28	(	0.99	-	1.66	)	1.15	(	0.93	-	1.42	)	1.11	(	0.95	-	1.29	)
*Parental education level*																		
Less than or some college or university	1.31	(	0.89	-	1.92	)	1.47	(	1.07	-	2.01	)	1.60	(	1.27	-	2.01	)
College diploma	1.33	(	0.92	-	1.93	)	1.37	(	1.01	-	1.86	)	1.42	(	1.14	-	1.77	)
University degree	1.39	(	0.99	-	1.94	)	1.14	(	0.87	-	1.50	)	1.29	(	1.06	-	1.56	)
*Income adequacy*																		
Lowest income adequacy	1.44	(	0.94	-	2.20	)	1.24	(	0.86		1.78	)	1.46	(	1.12	-	1.90	)
Lower middle income adequacy	1.52	(	1.07	-	2.16	)	1.56	(	1.18	-	2.08	)	1.07	(	0.85	-	1.35	)
Upper middle income adequacy	1.52	(	1.15	-	2.00	)	0.89	(	0.69	-	1.15	)	1.13	(	0.94	-	1.34	)
***Home environmental factors***																		
*Crowding (No. of people in household)*																		
4 people in household	1.22	(	0.88	-	1.70	)	0.86	(	0.66	-	1.12	)	1.03	(	0.85	-	1.26	)
≥ 5 people in household	1.16	(	0.83	-	1.64	)	0.78	(	0.59	-	1.03	)	0.93	(	0.75	-	1.15	)
Use of gas to cook or heat	0.98	(	0.77	-	1.26	)	0.82	(	0.67	-	1.01	)	0.99	(	0.85	-	1.16	)
Environmental tobacco smoke exposure	1.22	(	0.83	-	1.78	)	0.81	(	0.57	-	1.16	)	1.06	(	0.83	-	1.36	)
Pets	0.74	(	0.58	-	0.94	)	0.77	(	0.63	-	0.94	)	0.89	(	0.77	-	1.02	)
Cockroaches	1.02	(	0.69	-	1.51	)	0.99	(	0.70	-	1.39	)	1.24	(	0.96	-	1.59	)
Mould	1.11	(	0.78	-	1.60	)	1.11	(	0.80	-	1.53	)	1.14	(	0.90	-	1.44	)

Abbreviations: HR = hazard ratio; CI = confidence interval; No. = number

*Note: All models for 1,284 participants in this study were adjusted for 16 covariates: 6 child factors, 4 parental/demographic factors and 6 home environmental factors. Child factors included: age, sex, low birth weight (< 2500 g), prematurity (child born within three weeks of due date), breastfeeding, and enrollment in childcare. Parental/demographic factors included: parental asthma and atopy, parental education level, and income adequacy. Home environmental factors included: crowding (number of people living in household) and home exposures during first year of life (use of gas to cook or heat, exposure to environmental tobacco smoke, pets, cockroaches, and mould in the home)

## Discussion

This study focused on asthma, allergic rhinitis, and eczema or atopic dermatitis as they are the most common childhood allergic diseases and have significant impact on those suffering from these conditions.[13–15] A recently published review by Dierick et al. provided an overview of the prevalence and socio-economic impact of allergic diseases including asthma, allergic rhinitis, and eczema.[16] Globally, asthma prevalence in adults ranges from 1 to 20%, allergic rhinitis ranges from 1 to 18%, and atopic dermatitis ranges from 2 to 10%.[16] While these prevalence estimates varied widely and mainly focused on adults, it should be acknowledged that the prevalence of allergic diseases in children is generally even higher. As indicated by Dierick et al.’s review, asthma, allergic rhinitis, and atopic dermatitis have serious impacts on quality of life, direct, and indirect costs like work productivity being negatively affected due to presenteeism.[16] Previously, we found a 17% increase in risk of developing asthma and an 8% increase in risk of allergic rhinitis for each IQR increase in exposure to total oxidants (ozone [O_3_] and nitrogen dioxide [NO_2_]) at birth.[17] We did not report a significant association between the onset of asthma in children and PM_2.5_. However, a subsequent study that examined the impact of PM_2.5_ components on morbidity in children living with asthma found that sulphate was significantly associated with approximately 130% and 40% increases in risk per IQR increment in concentration for all-cause hospitalization and emergency department visits, respectively, after adjusting for potential confounding factors.[18] Following the same study cohort, this epidemiological study further examined ROS concentrations generated in the human respiratory tract in response to inhaled pollutants including Cu and Fe in PM_2.5_. We found that exposure to ROS at birth significantly increased the risk of asthma by 11%. These findings are important because they examined the effects of early life exposures to air pollution and their subsequent influence on health throughout childhood to adolescence. To our knowledge, our study is the first longitudinal epidemiological study that quantified the impact of exogenous ROS on health risks in children. Similar to our findings, Zhang et al. reported that ROS, but not Fe or Cu, was significantly associated with risk of incident asthma in adults based on an administrative cohort in Toronto.[19] Strengths of our study include the rich data on individual level covariates and long follow-up period. There are also some study limitations. Firstly, ROS is potentially spatially and temporally variant, however, we lack seasonal data to account for seasonal variation. Secondly, we do not have measurements of endogenous levels of ROS in our study population to account for the total impact of ROS on health. Next, the ROS data collected were limited to summer 2016 and winter 2017. We assumed that spatial patterns of ROS during this period were the same as at the time of the participants’ births. While there is evidence that spatial patterns of other traffic-related pollutants are stable over time periods of up to 12 years, this assumption likely introduces exposure measurement error.[20, 21] Lastly, all covariates in this study are parent-reported and may be incorrect. Future research incorporating epigenetics data in risk assessment and other endpoints such as health services use may be important to consider to better understand the total burden of ROS exposures in human populations.

## Conclusion

In conclusion, we found that ROS exposure in early life significantly increased the childhood risk of asthma, but not allergic rhinitis and eczema. Asthma may be a more sensitive measure of ROS impact than other allergic diseases. This study adds to the limited but growing body of literature on air pollution, specifically ROS, and adverse childhood allergic conditions and respiratory health outcomes, while using a birth cohort linked with large population-based health care utilization datasets and physician-diagnosed outcome measures.

## Data Availability

The data that support the findings of this study are housed at and are available from ICES, Ontario but their privacy and security policies govern the availability of these data. Contact ICES, Ontario for more information.

## Abbreviations

ROS: reactive oxygen species
PM_2.5_: fine particulate matter
ELF: epithelial lining fluid
T-CHEQ: Toronto Child Health Evaluation Questionnaire
ISAAC: International Study of Asthma and Allergies in Childhood
HCN: Health Card Number
ICD: International Classification of Disease
Fe: iron
Cu: copper
KM-SUB-ELF: kinetic multilayer model of surface and bulk chemistry in the lung epithelial lining fluid
PH: proportional hazards
HR: hazard ratios
CI: confidence interval
IQR: interquartile range
SD: standard deviation
O_3_: ozone
NO_2_: nitrogen dioxide
